# Microbiological and virulence aspects of *Rhodotorula mucilaginosa*

**Published:** 2020-05-27

**Authors:** Isabele Carrilho Jarros, Flávia Franco Veiga, Jakeline Luiz Corrêa, Isabella Letícia Esteves Barros, Marina Cristina Gadelha, Morgana F. Voidaleski, Neli Pieralisi, Raissa Bocchi Pedroso, Vânia A. Vicente, Melyssa Negri, Terezinha Inez Estivalet Svidzinski

**Affiliations:** 1 Division of Medical Mycology, Teaching and Research Laboratory in Clinical Analyses – Department of Clinical Analysis of State University of Maringá, Paraná, Brazil; 2 Postgraduate Program in Microbiology, Parasitology, and Pathology, Biological Sciences, Department of Basic Pathology, Federal University of Parana, Curitiba, Brazil; 3 Department of Dentistry, State University of Maringá, Maringá, Paraná, Brazil

**Keywords:** Rhodotorula mucilaginosa, characterization of biofilm, Tenebrio molitor

## Abstract

We aimed to characterize microbiologically clinical isolates of *R. mucilaginosa* isolated from colonization of a patient with chronic renal disease (CKD), as well as to evaluate their phylogeny, antifungal susceptibility, virulence, and pathogenicity in order to infer the potential to become a possible infective agent. For this study, two isolates of* R. mucilaginos*a from oral colonization of a CKD patient were isolated, identified and characterized by classical (genotypic and phenotypic) methods. Susceptibility to conventional antifungals was evaluated, followed by biofilm production, measured by different techniques (total biomass, metabolic activity, colony forming units and extracellular matrix quantification). Finally, the pathogenicity of yeast was evaluated by infection of* Tenebrio molitor* larvae. All isolates were resistant to azole and sensitive to polyenes and they were able to adhere and form biofilm on the abiotic surface of polystyrene. In general, similar profiles among isolates were observed over the observed periods (2, 24, 48 and 72 hours). Regarding extracellular matrix components of biofilms at different maturation ages, *R. mucilaginosa *was able to produce eDNA, eRNA, proteins, and polysaccharides that varied according to time and the strain. The death curve *in vivo* model showed a large reduction in the survival percentage of the larvae was observed in the first 24 hours, with only 40 % survival at the end of the evaluation. We infer that colonization of chronic renal patients by *R. mucilaginosa *offers a high risk of serious infection. And also emphasize that the correct identification of yeast is the main means for an efficient treatment.

## Introduction

Human fungal infections by *Rhodotorula *spp. are increasing in the last decades (Ioannou et al., 2018[18]), in China they are among the main causes of invasive fungal infections by non-candida yeasts (Xiao et al., 2018[51]) and are considered an emerging pathogen. It was classified as the third most commonly isolated yeast from blood cultures and the most common microorganism isolated from the hands of hospital employees and patients (Gomez-Lopez et al., 2005[16]). 

Species from this genus have been considered an opportunistic pathogen since they affect mainly immunocompromised individuals. A recent systematic review (Ioannou et al., 2018[18]) shows fungal infections by *Rhodotorula *spp. consist mainly of bloodstream infections, as well as central nervous system (CNS), affecting especially patients under the use of central venous catheters (CVC). However, there are reports in the literature proving that *Rhodotorula *spp. causes besides fungemia and meningitis, also cutaneous infections, peritonitis, keratitis, ventriculitis, ocular and other less frequent infections (Mohd Nor et al., 2015[27]; Fernández-Ruiz et al., 2017[12]; Franconieri et al., 2018[13]). These clinical presentations, as well as microbiological aspects, makes *Rhodotorula *infections look like cryptococcosis and to worsen this scenario, yeasts of this genus are usually azoles resistant, including isavuconazole, which has good *in vitro* activity against *Cryptococcus *species, but it is not effective against *Rhodotorula *spp. (Desnos-Ollivier et al., 2019[10]). Reinforcing that the management of these two fungal infections needs to be well known.

Although, endocarditis by* Rhodotorula* spp. has already been related to immunocompetent patients (Loss et al., 2011[23]; Simon et al., 2014[37]) apparently without risk factor for opportunistic infection, invasive infections caused by this yeast are mainly associated with underlying immunosuppression. The most affected are patients with severe diseases such as leukemia, cancer or other solid tumors, lymphoproliferative disease, HIV, diabetes mellitus and submitted to various types of surgery (De Almeida et al., 2008[8]; Tuon and Costa 2008[44]; Miglietta et al., 2015[25]; Falces-Romero et al., 2018[11]). Recently some cases of endocarditis by *R. mucilaginosa* have been described in chronic kidney disease (CKD) patients (Damasco et al., 2014[7]; Simon et al., 2014[37]; Cabral et al., 201[1]7). Nevertheless, despite their importance, invasive *Rhodotorula* spp. infections are not well explained yet, few is known about the virulence potential of this yeast, and whether the dissemination process depends more of pathogenic merit of the fungus or the patient debility. 

The association of *Rhodotorula* spp. with CVC or other invasive medical devices is justified by its ability of biofilm production (Ioannou et al., 2018[18]). These devices provide appropriate surfaces for biofilms formation and establishment. However, in contrast to the extensive literature on biofilms of *Candida *species, little attention has been paid to emerging fungal pathogens, such as *Rhodotorula* species (Melo et al., 2011[24]; Nunes et al., 2013[32]).

The biofilm installation at some surfaces contacted to the host can trigger an acute fungemia and consequently a disseminated infection. This occurs when the clusters of cells are dispersed from the initial biofilm and occupy a not colonized niche (Gulati and Nobile, 2016[17]). Recent studies have shown that cells that detach themselves from a biofilm have a greater association with severe infections, with high mortality rates compared to microorganisms in their planktonic form. In fact, more than 65 % of human infections involve the formation of biofilms, keeping up to the growing number of immunocompromised patients. In addition, more than 500,000 deaths per year are caused by biofilm-associated infections (Sardi et al., 2014[36]). 

*Rhodotorula *yeasts are part of the human microbiota as commensal microorganisms of skin, nails, gastrointestinal, urinary, and respiratory tracts. They are also widely found in nature and have been isolated from environmental sources, like air, soil, and plants (Chaud et al., 2016[3]). Falces-Romero et al. (2018[11]) isolated *R. mucilaginosa* from blood cultures of eight patients, six of them had a real infection and two were considered contaminants, since usually the yeasts causing infection, are from environmental origin or even from the microbiota itself (Mohd Nor et al., 2015[27]; Fernández-Ruiz et al., 2017[12]; Franconieri et al., 2018[13]). Recently, we showed that *R. mucilaginosa* was able to colonize and crossed a device used for dermis regeneration of burned patients in three days increasing significantly in seven days, therefore offering high risk for systemic infection (Jarros et al., 2018[19]). On that occasion, we demonstrated that commensal yeasts, commonly found in the environment, skin or mucosa of health professionals and patients, could offer a risk of infection for severe patients. In order to improve the knowledge about this question, we intend to expand the study addressed just to *R. mucilaginosa. *Thus, here we aimed to characterize microbiologically clinical isolates of* R. mucilaginosa* isolated from colonization of chronic renal patients as well as to evaluate some of the aspects related to phylogeny, antifungal susceptibility, virulence, and pathogenicity in order to infer the potential to become a possible infection.

## Materials and Methods

### Studied group and isolation

A project involving 243 patients with CKD, under the care of the nephrology service of a reference hospital in the northwest of Parana State, Brazil, between October and November 2014 was developed. For the purpose of this study, a patient, who was colonized by *Rhodotorula *spp. was selected. This voluntary is a man, 55 years old, diabetic (*Diabetes mellitus* type II), confirmed with chronic kidney diseases (stage 5) at 2 years before, and he was under hemodialysis for 6 months, no use of antifungals and absence of oral lesions. The data collection, the oral mucosa examination, were performed according to Pieralisi et al. (2016[34]). This study was conducted according to the Resolution 466/2012 of the National Health Council and was previously approved by the Ethics Committee for the Research Involving Humans of the State University of Maringá, Brazil [COPEP- EMU nº 383979, CAEE resolution nº 17297713.2.0000.0104]. 

### Microorganisms

This study was conducted with two clinical isolates from oral colonizations of one CKD patient plus the* R. mucilaginosa* ATCC 64684. For the clinical isolates the collecting biological samples and the cultivation method were performed as described previously (Pieralisi et al., 2016[34]). Briefly, yeasts were subcultured in chromogenic medium CHROMagar™ *Candida* (Difco, USA), to check the culture purity. The isolates were identified by classical tests, including macro and micro morphologies, fermentation tests and assimilation of carbohydrate and nitrogen sources (Statzell-Tallman and Fell 1998[41]; Larone, 2011[22]). To confirm the identification, mass spectrometry assisted by flight time desorption/ionization matrix (MALDI TOF-MS) was performed. For the MALDI TOF-MS method, the yeasts were prepared according to specific protocols (Pascon et al., 2011[33]; Spanu et al., 2012[40]) with a Vitek MS mass spectrometer using the Myla or Saramis software for data interpretation.

These yeasts were deposited at Microbial Collections of Paraná Network- TAX online and on Micoteca of the Medical Mycology laboratory, Laboratório de Ensino e Pesquisa em Análises Clínicas of Universidade Estadual de Maringá (LEPAC), with the identification codes: CMRP3462 (MK453051; Genbank) and CMRP3463 (MK453052; Genbank)*. *On LEPAC, the yeasts were stored in Sabouraud Dextrose Broth (SDB; Difco™, USA) with glycerol at –80 °C. All samples were cultured on SDA with additional chloramphenicol (0.1 %) and incubated at 25 °C for up to 3 days, after all tests (Moț et al., 2017[26]).

### Morphological characterization

The morphology was assessed with by optical microscopy (EVOS™ FL, Life Technologies) and by Scanning Electron Microscopy (SEM; Quanta 250™, ThermoFisher). The colony, cell morphology and the polysaccharide capsule were observed by light microscopy at 40x magnification. The colony was observed after microcultivation and analyzed directly by light microscopy (Larone, 2011[22]). To analyze capsule, a suspension of 500 µg/mL phosphate-buffered saline 0.01 mol/L, pH 7.4 solution (PBS) with one isolate colony and 500 µL of China ink was prepared and placed on a slide for observation under light microscope. The cell morphology was performed with Calcofluor White (Fluka Analytical, Canada) diluted in a proportion of 1:4 in PBS for 5 minutes, and excess dye was removed by washing once with PBS. The yeasts were observed with a filter capable of detecting the yeast cell wall (BP 365–370, FT 400, LP 421). SEM analysis was performed at Laboratory of Electron Microscopy and Microanalysis, Universidade Estadual de Londrina, Londrina, Paraná, Brazil, supervised by Admilton G. de Oliveira, according to Negri et al. (2011[30]). The samples were observed at 5000×magnification.

### Genotypic characterization (sequencing and phylogenetic study)

The DNA extraction of isolates was performed according Vicente et al. (2008[47]), using a silica: celite mixture (silica gel H, Merck 7736, Darmstadt, Germany/Kieselguhr Celite 545, Machery, Düren, Germany, 2:1, w/w). The internal transcribed region (ITS) was amplified using the universal primers ITS1 (5'-TCCGTAGGTGAACCTGCGG-3') and ITS4 (5'-TCCTCCGCTTATTGATATGC-3') (White et al., 1990[50]; Vicente et al., 2008[47]). PCR was performed in a 12,5 μL volume of a reaction mixture containing 4,3 μL of mix solution containing 0,3 mM dNTPs, 2,5 mM MgCl2, 1,25 μL reaction buffer, 0,5 μL of each primer (10 pmol) and 1 μL rDNA (20 ng/μL). The sequencing was performed by Sanger method in automated sequencer ABI3730 (Applied Biosystems Foster City, U.S.A). Consensus sequences of the ITS region were inspected using MEGA v.7 software and alignments were performed using MAFFT interface (online). The identification of species was determined by phylogenetic analysis, using type strains established by Wang et al. (2015[49]) and Nunes et al. (2013[32]). Phylogenetic tree was performed MEGA v.7 software with 1,000 bootstrap replicates using the maximum likelihood function and the best evolutionary model corresponding to the data set used. Bootstrap values equal to or greater than 80 % were considered statistically significant.

### Antifungal susceptibility profile

The antifungal susceptibility profile of *R. mucilaginosa *isolates was determined against amphotericin B (Sigma, USA), fluconazole (Sigma, USA), voriconazole (Sigma, USA), itraconazole (Sigma, USA) and nystatin (Sigma, USA). The test was performed by a microdilution assay in broth, according to the Clinical Laboratory Standards Institute (CLSI, 2008[5]), M27-A3 document. Concentrations ranged between 0.125 and 64 µg/mL for fluconazole, between 0.03 and 16 µg/mL for amphotericin B and voriconazole, between 0.0625 and 32 µg/mL for itraconazole and 0.25 and 128 µg/mL for nystatin. Suspensions were tested with antifungal solutions in 96-well microplates (Nunclon Delta; Nunc) incubated for 48 hours at 25 ºC. *C. albicans* ATCC® 90028 was used as a control and the reading of microplates was performed at 405 nm (Expert Plus Microplate Reader; ASYS). The Minimum Inhibitory Concentration (MIC) was determined according to CLSI, M27-A3. Results were given as: susceptible (S); susceptible dose-dependent (SDD); and resistant (R). Cut-off points were: S ≤ 8 µg/mL; SDD = 16–32 µg/mL; and R ≥ 64 µg/mL for fluconazole, S ≤ 1 µg/mL; SDD = 2 µg/mL; R ≥ 16 µg/mL for voriconazole, S ≤ 0.125 µg/mL; SDD 0.25-0.5 µg/mL; R ≥ 32 µg/mL for itraconazole, S ≤ 4 µg/mL; SDD = 8–32 µg/mL; R ≥ 64 µg/mL for nystatin. For amphotericin B, resistant isolates were defined as isolates with MIC > 1 µg/mL (Montagna et al., 2014[28]).

### Adhesion and biofilm formation assays

The biofilm formation assay was adapted from previously described method (Nunes et al., 2013[32]). The strains initially cultured in SDA at 25 °C for 72 hours were further subcultured into SDB and grown for 18 hours with shaking at 110 rpm at 25 °C. The grown cultures were harvested, washed twice with phosphate-buffered saline (PBS; pH 7.2), and adjusted to a concentration of 1x10^7^ cells/mL, using a Neubauer chamber in RPMI 1640 medium (Roswell Park Memorial Institute, Gibco). Biofilm formation was tested in sterile 96-well polystyrene flat-bottom plates (TPP®, Trasadingen, Switzerland) with 200 μL of inoculum. A test medium without yeasts was performed and used as a negative control. The plates were then incubated with agitation at 110 rpm at 25 °C for 2 hours. After this time, the supernatant was gently removed from the wells, the cells were washed three times with PBS for removal of non-adherent yeasts, 200 μL of RPMI 1640 were added and the plates were incubated at 110 rpm at 25 °C for 24, 48 and 72 hours. The processes of biofilm formation was evaluated after the adhesion (2 hours) and the different ages of biofilm maturation (24, 48 and 72 hours).

### Biofilm characterization

The adhesion and biofilms were analyzed by number of cultivable cells determined by counting colony forming units (CFU); metabolic activity by the tetrazolium salt 2,3-bis(2-methoxy-4-nitro-5-sulfophenyl)-5-(phenylamino)-carbonyl-2H-tetrazoliumhydroxid and (XTT; Sigma-Aldrich, USA) reduction assay; total biofilm biomass by crystal violet staining (CV); quantification of proteins, polysaccharides, extracellular DNA (eDNA), extracellular RNA (eRNA) in biofilm matrix using spectrophotometer; and finally biofilm structure by SEM. 

Briefly, the wells with different ages of biofilm (2, 24, 48 and 72 hours) were washed twice in PBS to remove loosely attached cells. Before determining the CFU, the time and potency of sonication were optimized in order to allow the complete removal of the adhered cells without causing any damage. After washing each well, the biofilms were resuspended with 100 μL of PBS and scraped. The suspensions were transferred a new tub and sonicated (Sonic Dismembrator Ultrasonic Processor, Fisher Scientific) for 50 seconds at 30 %, and then the suspension was vortexed vigorously to disrupt the biofilm matrix and serial decimal dilutions, in PBS, were plated onto SDA. Agar plates were incubated for 48 hours at 25 °C, and the total CFU per unit area (Log CFU/cm^2^) of microtiter plate well were enumerated.

The determination of metabolic activity and total biomass were evaluated after washing each well, according to Negri et al., 2016[29]. The absorbance values 492 nm to XTT assay and 620 nm to CV assay were standardized per unit area of well (absorbance/cm^2^). The absorbance values of the negative control wells were subtracted from the values of the test wells to account for any background absorbance (Negri et al., 2016[29]).

For the analysis of matrix compounds, the matrix of different ages of maturation (24, 48 and 72 hours) was extracted using a protocol described by Capoci et al*.* (2015[2]) with some modifications. In brief, the biofilm samples were scraped from the 24-well plates, resuspended with PBS, and sonicated for 50 seconds at 30 %, and then the suspension was vortexed vigorously. The suspension was centrifuged at 4000×g for 10 minutes, and the supernatant was filtered through a 0.22 μm nitrocellulose filter (Merck Millipore, Ireland) and stored at −20 ºC until analysis. Proteins, polysaccharides, eDNA and eRNA were measured by NanoDrop spectrophotometer (NanoDrop 2000 UV Vis Spectrophotometer, Thermo Scientific, Wilmington, DE, USA). 

The morphological characteristic of *R. mucilaginosa* biofilm formation process (2, 24, 48 and 72 hours) was observed by SEM. For the SEM analysis, were performed according described previously. The samples were observed with a Quanta 250™ SEM (Thermo Fisher) at 2000×magnification.

### In vivo pathogenicity in model Tenebrio molitor

The evaluation of survival after infection of *Tenebrio molitor* larvae of approximately 100-200 mg using a total of 10 larvae per group. Three concentrations of inoculum with ATCC 64684 *R. mucilaginosa* were evaluated for standardization of the highest lethality inoculum: 1-3× 10^3^, 10^4^ and 10^5^ CFU in sterile PBS in aliquots of 5 μL were injected using a Hamilton syringe (701 N, 26's gauge, 10 μL capacity), into the hemocoel, the second or third sternite visible above the legs and the ventral portion. Negative control included sterile PBS. The larvae were placed in sterile Petri dishes containing rearing diet and kept in darkness at 25 °C. Mortality was monitored once a day for 10 days. To establish larvae death, according to de Souza et al. (2015[9]) we visually verified melanization and response to physical stimuli by gently touching them. 

With the standardized inoculum (1-3× 10^5^), we infected 10 larvae with each one of the clinical isolates in order to evaluate the virulence potential. 

### Statistical analysis

All tests were performed in triplicate, on three independent days. Data with a non-normal distribution were expressed as the mean ± standard deviation (SD) of at least three independent experiments. Significant differences among means were identified using the ANOVA test followed by Tukey’s multiple-comparison test. For *in vivo* pathogenicity, we were using Kaplan–Meier survival plots, according to de Souza et al. (2015[9]). The data were analyzed using Prism 8.1 software (GraphPad, San Diego, CA, USA). Values of p < 0.05 were considered statistically significant.

## Results

**Figure 1:**
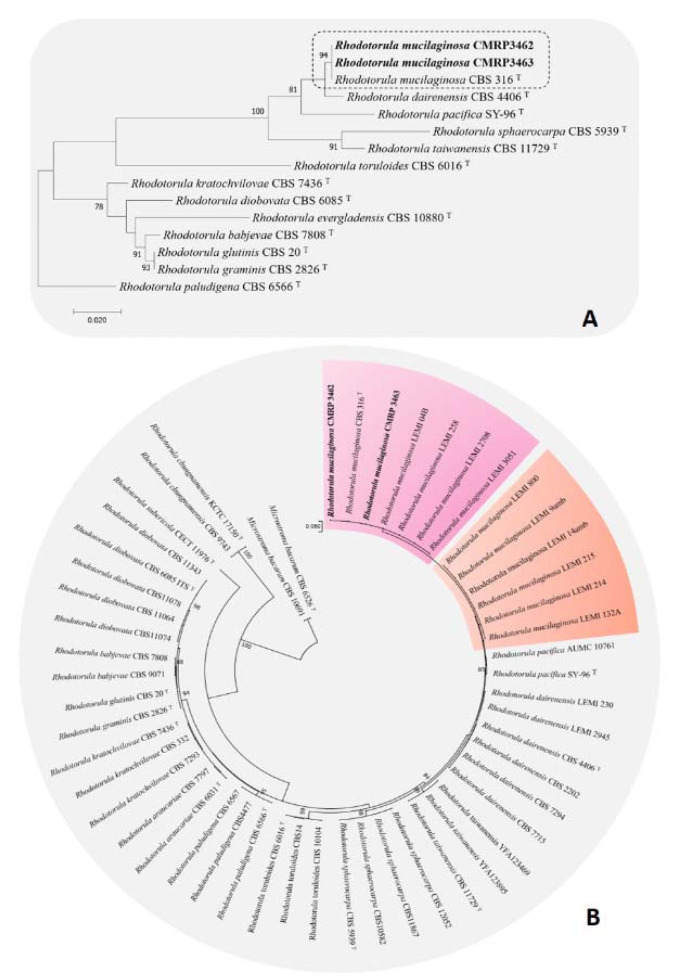
Phylogenetic analysis of *Rhodotorula mucilaginosa*, based on ITS sequences constructed with Maximum likelihood, based on the Tamura-Nei model + Gamma distribution (T92+G) implemented in MEGA v.7. Bootstrap support was calculated from 1000 replicates. (T) = type strain of the species. Bootstrap values > 80 % were considered statistically significant. (A) Phylogenetic tree of *Rhodosporidium* clade, *Sporidiobolaceae* family*. Rhodotorula paludigena *CBS6566T was taken as outgroup. (B) Phylogenetic tree of *R. mucilaginosa *variability among clinical and environmental isolates of Nunes et al. (2013). (amb) = environmental lineages.* Microstroma bacarum* CBS 6526T and CBS10691 were taken as outgroup.

Two clinical isolates obtained from saliva (CMRP3462) and sterile swab (CMRP3463) wiped in the center of the dorsal surface of the tongue collected were isolated and identified phenotypically by morphological plus biochemical aspects and confirmed as *R. mucilaginosa* by MALDI-TOF. In addition, the isolates were identified genotypically based on ITS region sequencing with following GenBank accession numbers CMRP3462 and CMRP3463 are MK453051.1 and MK453052.1. According to the phylogenetic analysis (Figure 1A(Fig. 1)), the isolates were located to the same clade of the type strain *R. mucilaginosa* CBS 316. The isolates were compared with clinical and environmental isolates from the other study (Nunes et al., 2013[32]) suggesting variability among the groups observed (Figure 1B(Fig. 1)).

**Figure 2:**
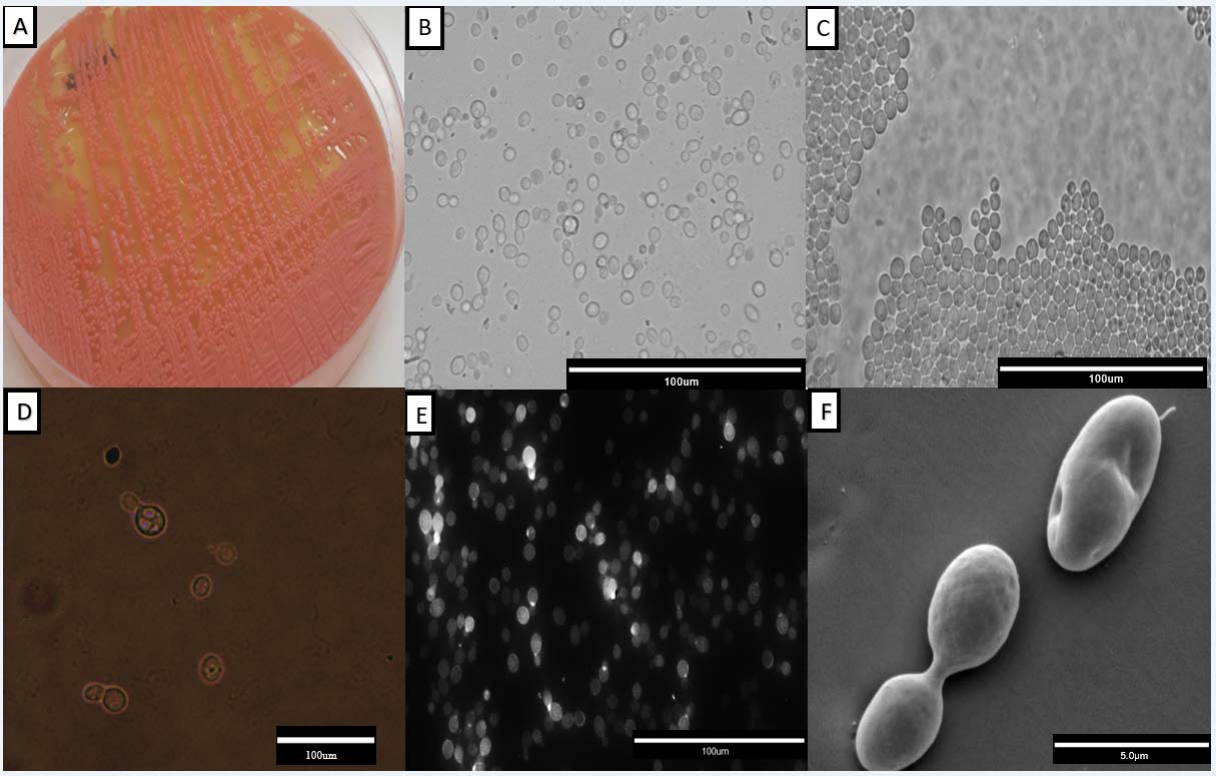
Representative morphological characterization of a *Rhodotorula mucilaginosa *isolate. In A, orange-colored mucoid colonies on Sabouraud Dextrose Agar; B, suspension of light field cells with a 40x magnification; C, characteristic microculture with rounded blastoconidias observed in a 40x magnification; D, polysaccharide capsule evidenced by China ink in 40x magnification; E, the cell wall evidenced by Calcofluor White; F, Scanning electron microscopy observed at 5000x magnification.

Observing cultures performed in SDA, we found orange-colored mucoid colonies (Figure 2A(Fig. 2)), which grew within 48 hours at 25 °C. In the microscopic examination, the round blastoconidia, without the rudimentary formation of hyphae, were observed (Figure 2B(Fig. 2)), as well as in the microculture (Figure 2C(Fig. 2)) to confirm the micromorphological characteristics. Using ink from China, it can be shown that *R. mucilaginosa* has a small polysaccharide capsule (Figure 2D(Fig. 2)), and Calcofluor White revealed the cell wall of this yeast, which turns out to be simple (Figure 2E(Fig. 2)). Finally, with SEM, we observed with more clarity the round blastoconidia, in division (Figure 2F(Fig. 2)).

All clinical isolates, as well as the ATCC isolate, were resistant to azoles (fluconazole, voriconazole, itraconazole), while for polyenes, amphotericin B and nystatin, all isolates were sensitive (Table 1(Tab. 1)).

**Figure 3:**
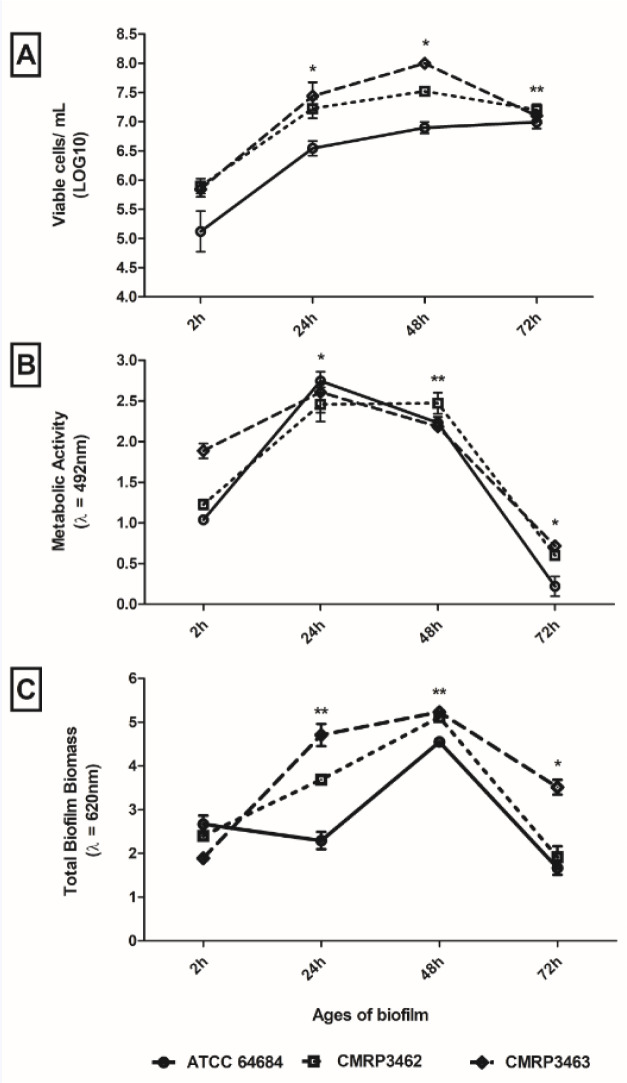
Adhesion capacity and biofilm formation, on polystyrene flat-bottom plates at different incubation times, of *Rhodotorula mucilaginosa*. A) Evaluation of cell viability by count of Colony Forming Units (CFU); B) Evaluation of metabolic activity by reduction of XTT; C) Evaluation of the production of extracellular matrix by Violet Crystal. *statistical difference in time among all isolates; **statistical difference over time for two isolates.

All the isolates of *R. mucilaginosa* showed the adhesion and biofilm formation abilities on the abiotic surface polystyrene. In general, similar profiles among the isolates were observed (Figure 3(Fig. 3)). There was a significant increase of biofilm in number of cells until 48 hours of biofilm age, after this period there is a decrease of viability cells (Figure 3A(Fig. 3)). On the other hand, metabolic activity and total biofilm biomass were different among the isolates, decreasing of 24 to 48 hours biofilm age (CMRP3463 and ATCC 64684) to metabolic activity (Figure 3B(Fig. 3)) and increasing from 24 to 48 hours biofilm age (CMRP3462 and ATCC 64684) to total biofilm biomass (Figure 3C(Fig. 3)). It is important to highlight that there was a decrease for all parameters analyzed after 48 hours of biofilm age.

Analyzing each isolate, regard to adhesion (2 hours), ATCC 64684 strain showed the lowest cell viability by CFU with p<0.01 (Figure 3A(Fig. 3)), the metabolic activity (XTT) was similar among the isolates (p>0.05) (Figure 3B(Fig. 3)). However, in the evaluation of total biofilm biomass (CV), according to Figure 3C(Fig. 3), the CMRP3463 was the lowest total biofilm biomass (p<0.01). From 24 hours of biofilm formation, clinical isolates (CMRP3462 and CMRP3463) were significantly (p<0.01) higher than ATCC 64684 to viable cells in biofilm (Figure 3A(Fig. 3)). All isolates presented a significant increase in relation 2 to 24 hours, with no statistical difference among isolates (Figure 3B(Fig. 3)) to metabolic activity. The clinical isolates (CMRP3462 and CMRP3463) increase significantly (p<0.01) in the total biofilm biomass at 2 to 24 hours, mainly CMRP3463 (Figure 3C(Fig. 3)). Finally, in the period of 48 to 72 hours, the clinical isolates (CMRP3462 and CMRP3463) showed a significant reduction of the number of cells viability (p<0.01). Further, there was a significant reduction for all isolates in metabolic activity and total biofilm biomass (p<0.01).

To the analysis of the extracellular matrix (ECM) of biofilms at different ages of maturation (24, 48 and 72 hours) eDNA, eRNA, proteins and polysaccharides were measured, as shown at Table 2(Tab. 2). *R. mucilaginosa* were able to produce ECM in different ages of biofilm constituted of eDNA, eRNA, proteins and polysaccharides. These matrix compounds varied according to the time and strain. For ATCC 64684, there was a significant increase of eDNA between 48 and 72 hours, whereas for CMRP3462 there was a significant reduction between these same times. On the other hand, CMRP3463 showed no differences in the amount of eDNA in the biofilms of 24, 48 and 72 hours. When eRNA was evaluated, there was a significant increase between 24 and 48 hours, which remained constant at 72 hours for ATCC 64684.

In relation to the clinical isolates CMRP3462 and CMRP3463, it was observed a contrary behavior, there is a greater amount of eRNA in 24 hours, while in 48 hours this amount is significantly lower and is maintained in 72 hours. For total proteins, there were no statistical differences among the isolates and the biofilm times evaluated. Finally, for polysaccharides we observed a significant increase at ATCC 64684 in 72 hours compared with the other ages of maturation of biofilm, while for the clinical isolates there were no statistical differences among the isolates and the biofilm times.

**Figure 4:**
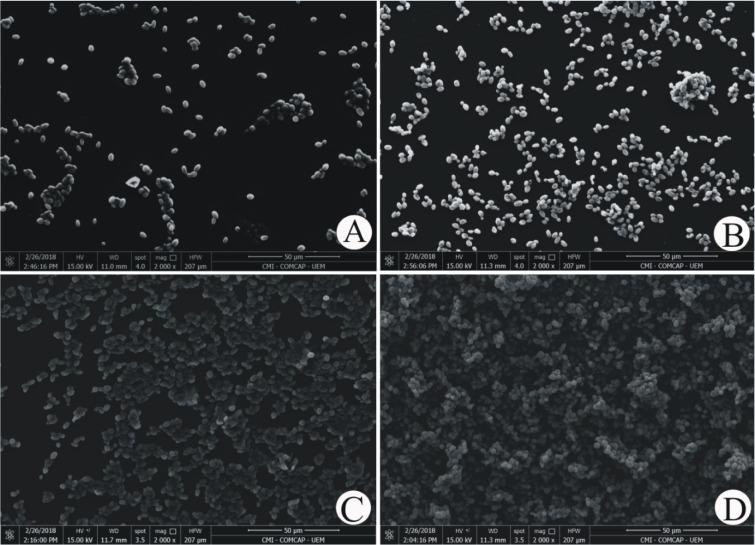
Illustrative images of ATCC 64684 *Rhodotorula mucilaginosa* adhesion and structure biofilms obtained by SEM, taken in a Quanta 250™ SEM (Thermo Fisher) magnification 2000×, shown different ages of maturation (24, 48 and 72 hours). A) The adherence at 2 hours of incubation; B) The biofilm at 24 hours; C) The biofilm at 48 hours; D) The biofilm at 72 hours.

Using light microscopy and scanning electron technology, it can be observed how the biofilm formation by *R. mucilaginosa*, which is shown in Figure 4(Fig. 4), proceeds.

In all situations,* R. mucilaginosa* cells were without filamentation, in blastoconidia form, uniform size and oval shape. From the time of adhesion to 2 hours of incubation (Figure 4A(Fig. 4)), one can find a few scattered fungal cells or small groups. After 24 and 48 hours, the yeasts were more clustered and in larger amounts, shown by Figure 4B and C(Fig. 4).

Finally, after 72 hours, it can be observed that the fully established biofilm with the confluence of *R. mucilaginosa* cells, as shown in Figure 4D(Fig. 4), is completely built up. With scanning electron microscopy (Figure 4D(Fig. 4)), several layers of cells can still be seen.

**Figure 5:**
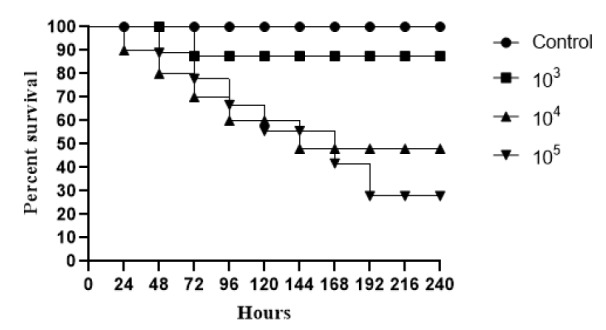
Survival curves of infected *Tenebrio molitor* with ATCC *Rhodotorula mucilaginosa*, for standardization. Groups of 10 larvae were infected with three fungal concentrations. Negative control group the *T. molitor* larvae were injected just with PBS (without yeasts).

In relation to the death curve, using *in vivo* Tenebrio* molitor* larvae model, three different inoculum concentrations for *R. mucilaginosa* ATCC 64684 (10^3^, 10^4^ and 10^5^) were evaluated. At the highest concentration (10^5^), we observed a reduction of approximately 60 % in the survival percentage of the larvae in relation to the control in 168 hours. At the lowest concentrations (10^3^ and 10^4^), we found approximately 80 % of survival at the end of the 10 days of evaluation (Figure 5(Fig. 5)). After defining the concentration 10^5^, we evaluated the clinical isolates, where we found that until the second day of evaluation, there was 15 % death for CMRP3462 and 20 % death for CMRP3463.

For more results see the Supplementary material.

## Discussion

*Rhodotorula *spp. is a common saprophytic fungus, being categorized as opportunistic and emerging pathogens recently (Ioannou et al., 2018[18]). An increase in the number of invasive infections by this yeast has been described in the last decades, with an overall mortality rate attributed around of 15 % (Tuon and Costa, 2008[44]). *R. mucilaginosa* is the most frequent species causing fungemia, which is responsible for up to 79 % of infections, followed by *R. glutinis* (7.7 %). These infections are often associated with the presence of CVCs or others implantable medical devices, and especially occur in immunocompromised individuals (De Almeida et al., 2008[8]; Loss et al., 2011[23]; Mohd Nor et al., 2015[27]; Jarros et al., 2018[19]).

*Rhodotorula* is a polyphyletic nature group (Wang et al., 2015[49]). Most species are environmental species, the family Sporidiobolale comprising the type species *R. glutinis*, an opportunistic species, and the emerging pathogen *R. mucilaginosa* (Figure 1(Fig. 1)). Besides *Rhodotorula *species are distributed among the family of *Pucciniomycotina phylum* (Wang et al., 2015[49]).

Current study included two clinical isolates of *R. mucilaginosa* obtained from a CKD patient living in south of Brazil, phylogenetic analysis was performed with sequences deposited by Nunes et al. (2013[32]), who evaluated isolates from 51 clinical and eight environmental isolates recovered from 14 different Brazilian hospitals from 1995 to 2010. Our goal was to correlate our samples with the bank of those authors (complementary material) and no differences were found between clinical isolates from different human sites, or between clinical and environmental isolates, neither geographic differences, these data reinforce the ubiquitous character of *Rhodotorula *spp. Nevertheless, as only ITS regions were evaluated, more in-depth studies, such as multi-locus region analyzes would be interesting in order to confirm this possibility (Chowdhary et al., 2019[4]).

 Regarding to micromorphological characterization we draw attention to a unique pattern of images displayed by *R. mucilaginosa*, found in all methodologies for both as in their planktonic forms (Figure 2(Fig. 2)) as in formed biofilms (Figure 4(Fig. 4)). Figure 2(Fig. 2) shows the coral-red-color colonies characteristic of the genus *Rhodotorula*, rounded blastoconidia, with absence of filamentation and presence of polysaccharide capsule, these characteristics are similar to those described by Gan et al*.* (2017[14]) and Kitazawa et al. (2018[20]).

The low morphological variation could also hinder the laboratory diagnosis in routine laboratories. Important to note that, in current study clinical isolates of *R. mucilaginosa* were correctly identified by morphological characterization together with the biochemical identification tests (assimilation and fermentation). These are simple and inexpensive tests, known by all laboratories and they were sufficient to identify this species, confirmed through MALDI-TOF and later through molecular techniques. In fact, macromorphology, especially the typical color exhibited by their colonies on SDA and micromorphology on cornmeal-Tween 80 agar are considered key characteristics for the presumptive diagnosis of this genus (Nunes et al., 2013[32]).

At the same time, it is important to emphasize this observation that *Rhodotorula spp.* can be falsely identified with *Cryptococcus spp.*, as both microorganisms, apart from morphological similarity, are urease-positive, not capable of fermentation and can assimilate glucose, maltose, sucrose, galactose, xylose, raffinose and trehalose (Larone, 2011[22]). Despite recently, Yockey et al. (2019[52]) suggested that *R. mucilaginosa* cells possess differences in signaling pathways, cell wall composition and that their membranes are more susceptible to perturbations than those of *C. neoformans*, similarities are observed in fungi morphology as well as in relation to the clinical aspects of the diseases caused by these two genera. Therefore, in some cases modern techniques are required to differentiate these microorganisms, such as the reporting of George et al. (2016[15]). These authors found a CKD patient with an unhealed lesion on the right elbow, multiple biopsies suggested cryptococcal infection with necrotizing granulomas. Although, a panfungal PCR on a skin biopsy identified as *Rhodotorula *spp. Summarizing, the laboratory supplies an important and definitive approach for differential diagnosis between these important diseases. The correct diagnosis becomes indispensable, as according to Ioannou et al. (2018[18]), 16.2 % of the systemic infections by *Rhodotorula *spp. attacked the CNS, where this species is the second most common fungal agent, affecting especially immunocompromised individuals.

In this type of patients there is a past predisposition to CNS infections by *Cryptococcus spp. *which, despite the similarity to *Rhodotorula spp.* as discussed above, the antifungal agents of choice are different because Fluconazole is indicated for *Cryptococcus spp.* while yeasts of the genus *Rhodotorula *are resistant to this class of antifungals (Stone et al., 2019[42]). It is important to note that yeasts belonging to* Rhodotorula *genus have a high level of fluconazole resistance, a greater tolerance for itraconazole and susceptibility for amphotericin B (Zaas et al., 2003[53]; Spader et al., 2019[39]). In agreement, we found in the antifungal susceptibility profile of *R. mucilaginosa* isolates resistant to azoles and sensitive to the other antifungals evaluated (Table 1(Tab. 1)). Recently, Wang et al.* (*2018[48]) have demonstrated that patients undergoing treatment with echinocandins addressed to other fungal infections have been reported to have fungal infections caused by *Rhodotorula* spp. since this yeasts are less susceptible to echinocandins due to the absence of 1,3-β-D-glucan in their cell walls.

In order to explain why this genus has, in addition to resistance to antifungal drugs, more widespread and severe infections in humans, it is possible that this yeast has putative virulence factors.

However, there are few studies in the literature that evaluate the virulence of *R. mucilaginosa* (Nunes et al., 2013[32]; Thomson et al., 2017[43]) thus, much information still is unknown.

Given the lack of filamentation capacity, it is important to understand what the mechanism of pathogenicity would be.

Biofilm production ability is one of the first suspicion due to the association of this genus with the use of CVC (Ioannou et al., 2018[18]). However, in contrast to the extensive literature dealing with biofilms of *Candida* spp. (Melo et al., 2011[24]; Gulati and Nobile, 2016[17]; Negri et al., 2016[29]), few studies on the biofilm produced by isolates of medical interest of *R. mucilaginosa *are available. This lack of knowledge deserves concern, since serious and fatal infections due to this species have been related to the formation of biofilms on medical devices (de Almeida et al., 2008[8]; Miglietta et al., 2015[25]).

The first study to evaluate the biofilm formation capacity of different *Rhodotorula* species (Nunes et al., 2013[32]) found differences between clinical and environmental isolates, through crystal violet staining only in 48 hours biofilms. Among the main results,* R. mucilaginosa* were classified as average biofilm producers, according to the classification scale for biofilm formation, adopted by the authors, which is in agreement to our results on biomass quantification.

The present study is the first one aiming to characterize the biofilm production by *R. mucilaginosa* on different ages (24, 48 and 72 hours), then we employed the classic methods used in studies with *Candida *spp. biofilms (Costa et al., 2013[6]; Negri et al., 2016[29]), that are CFU, CV, XTT and microscope, addressing to *R. mucilaginosa*. These results are presented on Figure 3(Fig. 3) and Table 2(Tab. 2) and complemented by the quantification of the extracellular matrix components determined on the same times (Table 2(Tab. 2)). It was possible to observe a formation and organization of the biofilm over time, with apex in 48 hours and probable dispersion at 72 hours, when there is decay of the ECM, metabolic activity and CFU. Although there is a general trend among isolates, it is possible to observe a weak difference in behavior at some specific times, suggesting characteristics isolate-dependent. In view of this scenario, we have concluded that the ECM of *R. mucilaginosa* is organized differently from other yeasts, such as *C. albicans*, because it has no visual filaments or other specialized structures, but its architecture resembles other pathogens that are not filamentous, such as *C. glabrata *and* Cryptococcus spp. *(Nobile and Johnson, 2015[31]).

On the other hand, when we see the biofilm architecture shown in Figure 4(Fig. 4), we hypothesized that in *R. mucilaginosa* a similar event occurs as in the biofilm of *Staphylococcus aureus*, where there is detachment of microcolonies of the biofilm and rolling up of these biofilm microcolonies (Rupp et al., 2005[35]). Thus, it would be possible that adherent cells as well as a part of the extracellular matrix were removed during the analysis steps. According to Rupp et al. (2005[35]) this is an important mechanism, mainly for non-motile microorganisms, as the controlled dispersal along surfaces in the protected biofilm state. Therefore, even so few information from Figure 3(Fig. 3) and Table 2(Tab. 2), biofilm by *R. mucilaginosa* would be important as infection source since detached yeast from biofilm would be in the same way being carried. Thus, is reasonable to assume that detached yeasts from mature biofilm on CVC could reach host cells and other sites of the human body by dissemination, as it has been previously described for *C. albicans* (Uppuluri and Lopez-Ribot, 2016[46]).

In fact, recent studies, with other pathogenic fungi, have shown the cells that detach themselves from a biofilm have a greater association with mortality, compared to microorganisms in their planktonic form. More than 65 % of human infections involve the formation of biofilms, which is related to the growing number of immunocompromised patients. In addition, more than 500,000 deaths per year are caused by biofilm-associated infections (Uppuluri et al., 2010[45]; Sardi et al., 2014[36]). Our results corroborate this idea, since according to Figure 3(Fig. 3), we found a high value of viable cells in biofilms, for all isolates of *R. mucilaginosa*, similar to that occurring in *C. albicans*.

In addition, we saw a peak of metabolic activity in 24 hours, and soon afterwards a decrease of this activity, being that in 72 hours, these cells were probably "dormant" (Negri et al., 2016[29]). Metabolically “dormant” yeast cells are also known as persistent cells, which originate stochastically as phenotypic variants within biofilms (Gulati and Nobile, 2016[17]). According to Kojic and Darouiche (2004[21]) persistent biofilm cells represent an important mechanism of resistance, and the eradication of a biofilm usually requires the administration of toxic concentrations of antimicrobials, and the recommended treatment includes removal of the contaminated device. Our findings could justify the association between *R. mucilaginosa *infections and biofilm formation.

SEM images reinforce our results on the biofilms evaluation, we observe that in the course of time, there was an increase in the number of cells and their organization. Unlike* C. albicans, C. parapsilosis or C. tropicalis*., we did not find filaments that give the structure for a complex biofilm, however we found several layers of cells, as well as described by Nunes et al. (2013[32]) and Soll and Daniels (2016[38]). With these variables, we infer that the maturity of *R. mucilaginosa* biofilm occurs in 48 hours due to stability and uniformity, confirmed mainly by microscopy images (Figure 4(Fig. 4)).

In view of the weak arguments that would justify the increase of *R. mucilaginosa *infections, we performed an *in vivo* infection on the invertebrate host *Tenebrio molitor, *an important tool to evaluate virulence of clinical pathogenic yeast strains (de Souza et al., 2015[9]). We have observed the development of these larvae and their resistance to external stimuli or situations. In our opinion, this is a model of a competent host, and it was fundamental because other *in vivo* studies found in the literature have been conducted with immunosuppressed animals (Thomson et al., 2017[43]). Here, *T. molitor* larvae were used, for the first time, with *R. mucilaginosa*, in order to evaluate the pathogenicity of this species. Surprisingly, the survival curves obtained (Figure 5(Fig. 5)) were similar to those found for *Cryptococcus neoformans* (de Souza et al., 2015[9]), a recognized human pathogen, which can be misidentified with *Rhodotorula *spp. regarding clinical and laboratory aspects (George et al., 2016[15]). Increasing concentrations of inoculum (10^5^) of this yeast resulted in high mortality rates, confirming the efficiency of the method to evaluate the virulence of pathogenic yeasts and showing, for the first time, the pathogenic potential of *R. mucilaginosa.* Although it is the highest fungal concentration tested in this study, these results indicate a high risk of infection and mortality also in humans.

## Conclusion

In this way, we infer that colonization of immunosuppressed patient by *R. mucilaginosa* offers a high risk of serious infection since this yeast showed highly pathogenicity for *in vivo* model, suggesting high risks of infection and lethality. Besides that, it is able to form biofilm on the surfaces of the medical devices, and apparently, the attached cells, as well as part of the extracellular matrix are removed and would fall into the circulatory chain being an important source of systemic infection. In addition, it is highly resistant to conventional antifungal agents, even antifungal of the last generation. We also emphasize that the correct identification of yeast is the main means for an efficient treatment.

## Acknowledgements

This study was supported by Coordenação de Aperfeiçoamento de Pessoal de Nível Superior – Brasil (CAPES) - Finance Code 001, Conselho Nacional de Desenvolvimento Científico e Tecnológico (CNPq) nº 421620/2018-8, Fundação de Amparo à Pesquisa do Estado do Paraná (Fundação Araucária) and Financiadora de Estudos e Projetos (FINEP/COMCAP).

The authors would like to thank the Laboratory of Electron Microscopy and Microanalysis, Universidade Estadual de Londrina (UEL/SETI).

## Declaration of interest statement

The authors declare that they have no conflicts of interest.

## Supplementary Material

Supplementary material

## Figures and Tables

**Table 1:**
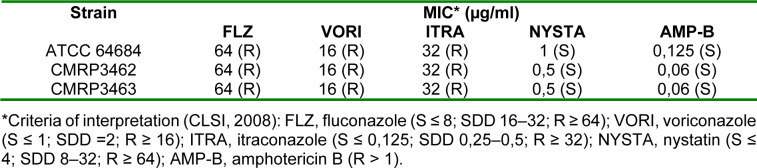
Antifungal susceptibility profile of the clinical isolates and the reference strain was determined for the following antifungals: fluconazole, voriconazole, itraconazole, nystatin and amphotericin B, according to the guideline of the Clinical Laboratory Standards Institute (CLSI, 2008), and M27-A3 document.

**Table 2:**
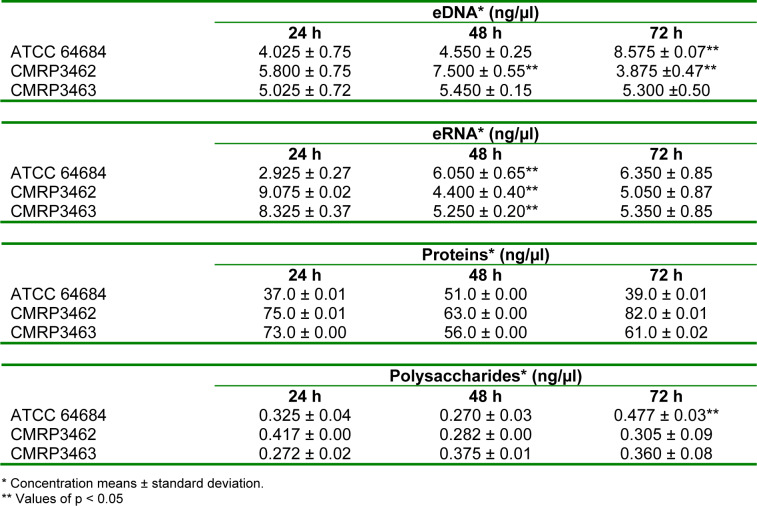
Quantification of extracellular DNA, extracellular RNA, proteins and polysaccharides for biofilm matrix analysis performed by NanoDrop spectrophotometer (NanoDrop 2000 UV Vis Spectrophotometer, Thermo Scientific, Wilmington, DE, USA).
