# Supplementary material for: Microbiological and virulence aspects of Rhodotorula mucilaginosa
Source: EXCLI J. 2020 May 27;19:687–704. (PMC7290102)
Supplement: Supplementary material [file EXCLI-19-687-s-001.pdf]

EXCLI Journal 2020;19:687-704 – ISSN 1611-2156
Received: October 16, 2019, accepted: May 25, 2020, published: May 27, 2020

Original article:

MICROBIOLOGICAL AND VIRULENCE ASPECTS OF *RHODOTORULA MUCILAGINOSA*

Isabele Carrilho Jarros[1], Flávia Franco Veiga[1], Jakeline Luiz Corrêa[1], Isabella Letícia Esteves Barros[1], Marina Cristina Gadelha[1], Morgana F. Voidaleski[2], Neli Pieralisi[3], Raissa Bocchi Pedroso[1], Vânia A. Vicente[2], Melyssa Negri[1], Terezinha Inez Estivalet Svidzinski[1*]

[1] Division of Medical Mycology, Teaching and Research Laboratory in Clinical Analyses – Department of Clinical Analysis of State University of Maringá, Paraná, Brazil
[2] Postgraduate Program in Microbiology, Parasitology, and Pathology, Biological Sciences, Department of Basic Pathology, Federal University of Parana, Curitiba, Brazil
[3] Department of Dentistry, State University of Maringá, Maringá, Paraná, Brazil

* **Corresponding author:** Terezinha Inez Estivalet Svidzinski, Division of Medical Mycology, Teaching and Research Laboratory in Clinical Analysis – Department of Clinical Analysis of State University of Maringá, Paraná, Brazil. Av. Colombo, 5790 CEP: 87020-900. Maringá, PR., Brazil. Phone: +5544 3011-4809, Fax: +5544 3011-4860, E-mail: tiesvidzinski@uem.br or terezinha.svidzinski@gmail.com

http://dx.doi.org/10.17179/excli2019-1672

This is an Open Access article distributed under the terms of the Creative Commons Attribution License (http://creativecommons.org/licenses/by/4.0/).

EXCLI Journal 2020;19:687-704 – ISSN 1611-2156
Received: October 16, 2019, accepted: May 25, 2020, published: May 27, 2020

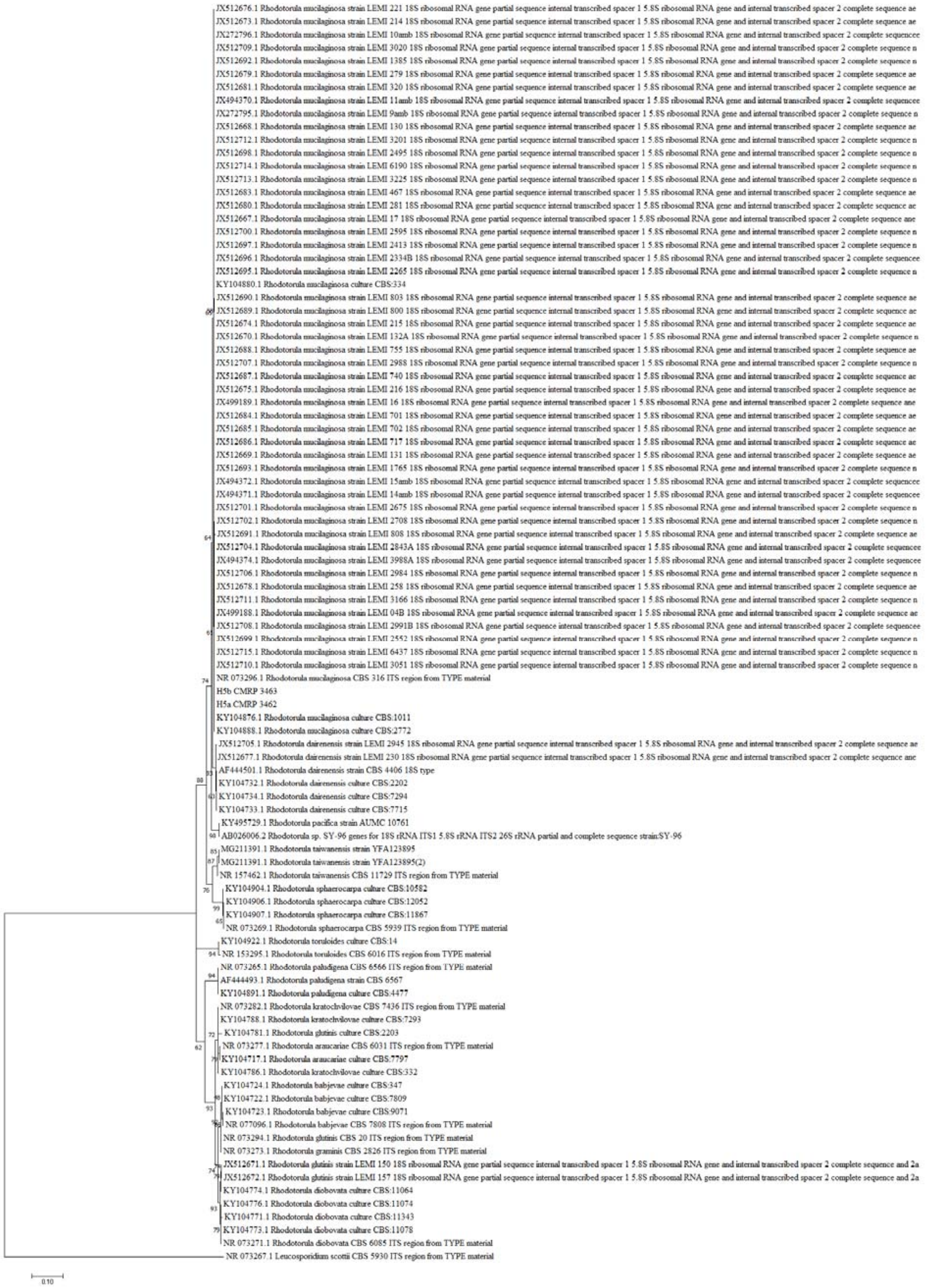

Supplementary Figure 1: Phylogenetic analysis of *Rhodotorula mucilaginosa*, based on ITS sequences constructed with Maximum likelihood, based on the Tamura-Nei model + Gamma distribution (T92+G) implemented in MEGA v.7. Bootstrap support was calculated from 1000 replicates. (T) = type strain of the species. Bootstrap values > 80 % were considered statistically significant.
